# Social skills in children with RASopathies: a comparison of Noonan syndrome and neurofibromatosis type 1

**DOI:** 10.1186/s11689-018-9239-8

**Published:** 2018-06-18

**Authors:** Elizabeth I. Pierpont, Rebekah L. Hudock, Allison M. Foy, Margaret Semrud-Clikeman, Mary Ella Pierpont, Susan A. Berry, Ryan Shanley, Nathan Rubin, Katherine Sommer, Christopher L. Moertel

**Affiliations:** 1 Division of Clinical Behavioral Neuroscience, Department of Pediatrics, University of Minnesota, 420 Delaware Street SE, Mayo Mail Code 486, Minneapolis, MN 55455 USA; 2 Division of Genetics & Metabolism, Department of Pediatrics and Ophthalmology, University of Minnesota, 2450 Riverside Avenue, Minneapolis, MN 55455 USA; 3 Biostatistics Core, University of Minnesota, 717 Delaware Street SE, Minneapolis, MN 55414 USA; 4 University of Minnesota Health, 2450 Riverside Avenue, Minneapolis, MN 55455 USA; 5 Division of Pediatric Hematology and Oncology, Department of Pediatrics, University of Minnesota, Mayo Mail Code 484, 420 Delaware Street SE, Mayo Mail Code 486, Minneapolis, MN 55455 USA

**Keywords:** RASopathies, Noonan syndrome, Neurofibromatosis type 1, NF1, Social, Neuropsychological, Language

## Abstract

**Background:**

Gene mutations within the RAS-MAPK signaling cascade result in Noonan syndrome (NS), neurofibromatosis type 1 (NF1), and related disorders. Recent research has documented an increased risk for social difficulties and features of autism spectrum disorder (ASD) among children with these conditions. Despite this emerging evidence, the neuropsychological characteristics associated with social skills deficits are not well understood, particularly for children with NS.

**Methods:**

Parents of children with NS (*n* = 39), NF1 (*n* = 39), and unaffected siblings (*n* = 32) between the ages of 8 and 16 years were administered well-validated caregiver questionnaires assessing their child’s social skills, language abilities, attention-deficit hyperactivity disorder (ADHD) symptoms and anxiety.

**Results:**

With respect to overall social skills, average ratings of children in both clinical groups were similar, and indicated weaker social skills compared to unaffected siblings. Although ratings of social skills were outside of normal limits for more than four in ten children within the clinical groups, most of the deficits were mild/moderate. Fifteen percent of the children with NS and 5% of the children with NF1 were rated as having severe social skills impairment (< − 2SD). Independent of diagnosis, having fewer ADHD symptoms or better social-pragmatic language skills was predictive of stronger social skills.

**Conclusions:**

Amidst efforts to support social skill development among children and adolescents with RASopathies, neuropsychological correlates such as social language competence, attention, and behavioral self-regulation could be important targets of intervention.

## Background

Noonan syndrome (NS) and neurofibromatosis type 1 (NF1) belong to a group of genetic syndromes caused by mutations affecting molecules within a common cellular signaling pathway known as the Ras-map kinase (RAS-MAPK) pathway. While each of these syndromes (“RASopathies”) has a unique phenotype, commonalities have been identified with regard to clinical features such as craniofacial differences, cardiac disease, skin abnormalities, developmental delay, and increased risk for malignancies [1]. NS and NF1 are the two most common and extensively studied RASopathies. NS is estimated to occur in approximately 1:1000 to 1:2500 births [2] and is typified by distinctive facial features, short stature, congenital heart disease, and skeletal anomalies, among other features. NS can be confirmed by laboratory testing identifying a mutation affecting one of several genes within the RAS-MAPK pathway. Mutations in the most commonly affected gene, *PTPN11*, account for approximately 50% of cases. Although pathogenic mutations causing NS have been found in approximately 15 different genes [3, 4], the etiology remains unknown among ~ 25% of individuals with a clinical diagnosis [5]. Mutations in particular locations on some RAS genes can also result in a distinctive phenotype; for example, some *PTPN11* mutations result in NS while others result in a NS-like presentation termed Noonan syndrome with multiple lentigenes (NSML). NF1 occurs in approximately 1:3000 births and is caused by mutations in the *NF1* gene, which encodes the protein neurofibromin [6]. NF1 is characterized by a set of symptoms including cutaneous features (e.g., café-au-lait macules, axillary freckling), neurofibromas, Lisch nodules, optic gliomas, and skeletal anomalies.

Recent research suggests that the shared mechanism of RAS-MAPK pathway dysregulation may lead to a pattern of similar behavioral and psychiatric comorbidities among RASopathy conditions. In particular, a higher prevalence of cognitive and learning disabilities, attention deficit hyperactivity disorder (ADHD), and characteristics of autism spectrum disorder (ASD) has been observed in both NS and NF1 [7–10]. Aligned with this finding, there is an expanding literature documenting overall poorer social competence (i.e., impairment in daily life skills that affect interpersonal interactions) in children with NS [11] as well as children with NF1 [12–14]. Although no studies have explored whether similar neuropsychological mechanisms underlie the heightened frequency of impaired social competence among children with these two biologically related genetic conditions, observations from our clinical experience working with children with NS and NF1 has suggested a high degree of overlap in the behavioral phenotype of the two most common RASopathies. Therefore, a first objective of this study was to compare informant ratings of social skill development in children with NS and NF1, in order to determine whether the frequency and severity of social skill impairment was comparable between the two clinical groups. We hypothesized that both clinical groups would exhibit impairment in social skills relative to a comparison group of unaffected siblings.

A second objective of the study was to determine whether similar neuropsychological features predict the social skills of children with different RASopathies. Past research has produced conflicting results with regard to which cognitive and psychological variables emerge as the best predictors of social competence. Limitations in social skills (e.g., difficulty showing concern for others, accepting differences, demonstrating friendship-seeking behaviors, managing conflict, or helping others) are commonly seen among individuals with mild to moderate intellectual disability [15, 16], suggesting that social competence may be closely linked with a child’s general level of developmental functioning. While intellectual disability is present in only a small minority of patients with NF1 and NS, there is an increased risk for other neurocognitive deficits and learning disabilities with these conditions [17, 18]. Hence, it might be assumed that some degree of social skills deficit relative to chronological age peers should be expected, given the higher rate of reasoning and learning problems in this population. Recent research studies have not supported this assumption. Multiple studies have failed to find a significant association between measures of social competence or ASD symptoms and global measures of intellectual functioning or academic achievement in children with NS and in those with NF1 [7, 12, 13, 19–21].

Given these previous findings, the current study focused on more specific neurocognitive attributes (i.e., language skills) and secondary comorbidities (i.e., ADHD and anxiety) that might be expected to impact social development in children with RASopathies. In both NS and NF1, problems with language and communication skills have been identified as a potential risk factor for poorer social functioning [22–24]. In terms of other behavioral health concerns, poorer social skills in children with NF1 have alternatively been found to be associated with symptoms of ADHD [13] or anxious behavior [12]. To clarify whether similar mechanisms underlie social skills deficits in these biologically related syndromes, we modeled the relative contributions of language/communication abilities, ADHD symptoms, and level of anxiety toward the capacity of children with NF1 and NS to demonstrate age-appropriate social skills in daily life.

## Methods

### Participants and procedures

Study participants were recruited from two outpatient clinics (a neurofibromatosis specialty clinic and a genetics clinic at the University of Minnesota; *n =* 55), at meetings of the Noonan Syndrome Foundation and Minnesota Neurofibromatosis Symposium (*n =* 27), and through patient listservs and social media sites (*n* = 28). Parents of individuals with NS or NF1 who had at least one child between the ages of 8 and 16 years were invited to participate. Participants provided written informed consent prior to completion of study questionnaires. The study was approved by the University of Minnesota Institutional Review Board.

Study questionnaires were completed by parents of 110 children and adolescents with NS or NF1 (Table 1). The mean age range of enrolled participants was 11.9 years (SD = 2.6 years; range 8.0 to 16.8 years). Exclusion criteria for the study included (a) children lacking a clinical diagnosis of NF1 *or* NS *or* a sibling diagnosed with one of these conditions and (b) significant sensory impairment. Data from one child with NF1 were excluded due to the patient’s severe vision loss. Diagnostic criteria for NS and NF1 were confirmed by a medical examination and/or review of clinical genetics reports. A maximum of two children per family were allowed to enroll in the study.

**Table 1:** Study cohort characteristics

	Noonan syndrome (*n* = 39)	NF1 (*n* = 39)	Unaffected siblings (*n* = 32)
Patient demographics	Mean (SD)	Mean (SD)	Mean (SD)
Child age at assessment	12.10 (2.70)	11.95 (2.50)	11.74 (2.54)
Parental years of education	15.90 (1.98)	15.17 (2.02)	14.94 (2.08)
	*N* (%)	*N* (%)	*N* (%)
Parent confirmed to be affected	4 (10%)	17 (44%)	5 (16%)
Gene mutations confirmed by laboratory testing			
NS/NSML-*PTPN11*	21 (54%)	–	–
NS-*SOS1*	4 (10%)	–	–
NS-*KRAS*	3 (8%)	–	–
NS-*RAF1*	2 (5%)	–	–
NS-*SHOC2*	2 (5%)	–	–
NS-*SOS2*	1 (3%)	–	–
NS-*MAP2K1*	1 (3%)	–	–
NF1-*NF1*	–	25 (64%)	–
Medical complications			
Preterm birth	14 (39%)	3 (9%)	1 (3%)
Cardiac disease	24 (62%)	1 (3%)	–
Seizures	4 (10%)	3 (8%)	–
Hydrocephalus	2 (5%)	0 (0%)	–
Chemotherapy treatment	0 (0%)	4 (10%)	–
Tumor/malignancy			
Optic pathway glioma	0 (0%)	3 (8%)	–
Other intracranial gliomas	0 (0%)	1 (3%)	–
Plexiform neurofibroma	0 (0%)	4 (10%)	–
Malignant peripheral nerve sheath tumor	0 (0%)	1 (3%)	–
Other neurofibromas	0 (0%)	4 (10%)	–

–Data not collected for this group

The cohort included 39 children and adolescents with NS (16 males, 23 females). Three children had been diagnosed with NSML (with confirmed *PTPN11* mutation) and were included with the NS group. The NF1 group consisted of 39 children (20 males, 18 females, 1 transgender individual) with confirmed diagnosis of NF1 based on the criteria established at the National Institutes of Health Consensus Conference [25]. Confirmation of the diagnosis by genetic testing was available in 34 individuals with NS and 25 children with NF1 (Table 1). The sibling comparison group consisted of 32 unaffected, typically developing individuals (17 males, 15 females). The two diagnostic groups and the sibling group did not differ significantly in chronological age, *F*_107_ = .172, *p* = .84, *d* = .11 (95% CI − 0.30 to 0.52).

### Measures

*SSIS*: The Social Skills Improvement System (SSIS) [26] is a parent/caregiver questionnaire measuring the frequency that a child demonstrates fundamental social competencies in everyday life (e.g., making friends, showing concern for others, resolving disagreements calmly, taking responsibility for their actions). This measure is frequently used by professionals to screen children suspected of having deficits in social behavior and to measure progress within therapeutic intervention. Raw scores on 11 subscales are combined to produce standardized scores in two summary categories: Social Skills (7 subscales: Communication; Cooperation; Assertion; Responsibility; Empathy; Engagement; Self-Control) and Problem Behaviors (4 subscales: Externalizing; Bullying; Hyperactivity/Inattention; Internalizing). The Social Skills composite score was used as our primary outcome measure of social competence. Higher scores on this scale indicate the presence of more positive social behaviors, thus a higher social competence. The SSIS parent form has adequate internal consistency reliability (Cronbach’s alpha = .75–.88 for the subscales) and test-retest reliability (.84 for the Social Skills composite) as well as moderate to high correlations with other widely used social skills measures.

*CCC-2:* The Children’s Communication Checklist, Second Edition (CCC-2) [27], measures both structural language abilities (e.g., speech intelligibility, grammatical and semantic knowledge) as well as pragmatic language abilities reflecting social context (e.g., nonverbal communication, appropriateness of topic, use of stereotyped language). This tool has demonstrated utility in identifying language impairment among children with a variety of neurodevelopmental disorders [28, 29]. Further, among children who were higher-functioning and had relatively strong structural language abilities, the CCC-2 has been found to be a more sensitive identifier of pragmatic language impairment than direct testing [30]. The primary summary score for the CCC-2 is the General Communication Composite (GCC). Researchers have also utilized separate summary scores for Structural Language (combining subscales A–D: speech, syntax, semantics and coherence) and Pragmatic Language (subscales E–H: initiation, scripted language, context and nonverbal communication) to assess function within these more specific domains [31].

*ADHD-RS:* The ADHD Rating Scale IV (ADHD-RS) [32], Home Version, is a widely used clinical research tool consisting of 18 items corresponding to each of the symptom criteria for ADHD in the Diagnostic and Statistical Manual of Mental Disorders, Fifth Edition (DSM-5) [33]. Nine items assess inattentive symptoms and nine items assess hyperactive/impulsive symptoms. Each symptom is rated on a scale of 0 (not present) to 3 (most severe).

*BASC-2:* The Behavior Assessment System for Children, Second Edition (BASC-2), is a well-validated broadband scale of emotional and behavioral functioning, which includes scales measuring various aspects of psychopathology (e.g., hyperactivity, depression, anxiety, aggressive behavior, conduct problems, somatic complaints) [34]. The Anxiety subscale was used to rate the presence of anxiety symptoms (e.g., worry, fear of mistakes, tension/nervousness, panic attacks) among study participants.

*Background History Form*: A demographic form was completed to obtain information about each child’s age, medical history, previous mental health diagnoses (Table 2), and family background.

### Statistical analysis

An ANOVA, followed by pairwise two-sample *t* tests, was used to compare mean total social skills scores (SSIS) between the NS, NF1, and sibling groups. Multiple linear regression was used to estimate the predictive effects of language (CCC-2; Pragmatic and Structural Language components), ADHD symptoms (ADHD-RS; Total score), and anxiety symptoms (BASC-2; Anxiety scale) on social skills as measured by the SSIS Social Skills composite score. Two models were fit, one with siblings included and one without, to evaluate whether predictive effects differed in NF1 or NS patients compared to healthy controls. Variables were pre-specified; however, a second set of models was fit which substituted the General Communication Composite (GCC) variable for the two language subscale variables. The effect of GCC was similar to that of the Pragmatic Language subscale. Exploratory analysis suggested that assuming a linear relationship between SSIS and other variables was reasonable. Analysis was performed with R software, version 3.4.

## Results

### Social skills

**Fig. 1:**
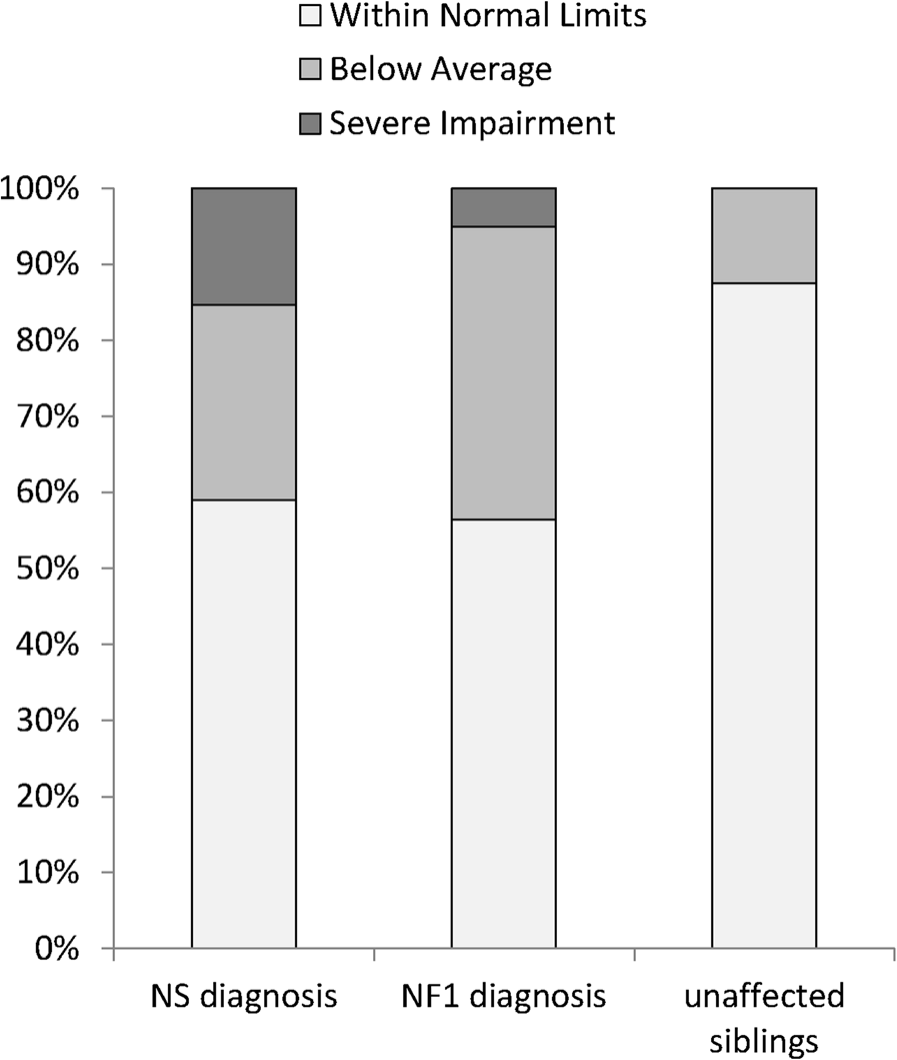
Parent ratings of social skills in children with NS, NF1, or unaffected siblings using the Social Skills Improvement System (SSIS)

Approximately half of the children and adolescents with RASopathies in this study were rated as having age-appropriate social skills on the primary outcome measure, the SSIS Social Skills composite (Fig. 1). Sixteen (41%) children with NS and 17 (44%) children with NF1 scored outside of normal limits. Most of these children showed a relatively mild social skills deficit. Ten (26%) children with NS and 15 (38%) children with NF1 scored in the below average range (between − 1 SD and − 2 SD) on this measure, as compared to four (13%) unaffected siblings who scored in the below average range. A smaller group of children demonstrated a severe deficit. Six (15%) of the children with NS and two (5%) participants with NF1 had scores indicating severe social skills impairment (− 2 SD or below), whereas none of the unaffected siblings had scores indicating severe social impairment.

**Table 2:** Frequency of previous intellectual, attention, or autism spectrum disorder diagnoses among study participants with RASopathies

		Estimated syndrome-wide prevalence (literature)		Parent-reported previous diagnoses (current cohort)	
		NS	NF1	NS	NF1
Intellectual disability (ID)	*n*	6–23% [18]	4–8% [8]	5/39	1/39
	%			13%	3%
Autism spectrum disorder (ASD)	*n*	15–30% [20, 52]	11–26% [21, 40, 53]	2/39	4/39
	%			5%	10%
Attention deficit hyperactivity disorder (ADHD)	*n*	22–48% [11, 20, 54]	30–50% [55]	10/39	15/39
	%			26%	38%

A one-way ANOVA revealed significant differences among the RASopathy and comparison groups on the Social Skills composite (*p* = 0.00045). When directly comparing the social competence of individuals in the RASopathy groups, children in the NS group were rated as having similar social skills to the NF1 group [mean difference = − 1.4; 95% CI − 5.5, 8.3; *p* = 0.690]. In contrast, children in both RASopathy groups were rated as having significantly poorer social skills relative to unaffected siblings [NS vs. siblings: mean difference = − 13.2; 95% CI − 20.1, − 6.3; *p* < 0.001] [NF1 vs. siblings: mean difference = − 11.8; 95% CI − 18.4, − 5.2; *p* < 0.001]. Descriptive statistics for the three groups on the SSIS scales are reported in Table 3.

**Table 3:** Parent ratings on a measure of social skills

		NS	NF1	Unaffected siblings
		(*n* = 39)	(*n* = 39)	(*n* = 32)
SSIS—Social Skills	Mean	87.62	89.00	100.81
	SD	16.01	14.44	13.31
	Range	54–117	57–116	77–125
SSIS—Problem Behaviors	Mean	115.28	114.28	101.19
	SD	16.47	14.15	13.05
	Range	85–154	92–148	82–128

*SSIS* Social Skills Improvement System

The relationship between social skills impairment and parent-reported previous mental health diagnoses of intellectual disability, autism spectrum disorder, and ADHD was examined (Table 2). All eight children with RASopathies who were rated as having severe social skills impairment (70 or below on the SSIS) had a previous diagnosis of ADHD. One of these eight children also had a diagnosis of an intellectual disability, and one had a diagnosis of autism spectrum disorder. Among participants with RASopathies who had been diagnosed with intellectual disability (*n* = 6), mean (SD) score on the SSIS Social Skills composite was 90.50 (19.41). Among those with an autism spectrum disorder diagnosis (*n* = 6), the mean (SD) SSIS score was 83.17 (9.83).

Given some research suggesting that social impairment may be less apparent in younger children with RASopathies, or that individuals may show declining performance relative to peers as they grow older [35, 36], we examined the correlation between age and social skills in each of the groups. No association between age and SSIS Social Skills scores was found in the NS group, *r* = − 0.04, *p* = 0.787 or the sibling comparison group, *r* = 0.14, *p* = 0.452. For the NF1 group, the correlation of age and social skills suggested that older children with NF1 in this cohort had slightly better social skills, *r* = 0.30, *p* = 0.060, although this finding was not statistically significant. Overall, there was no evidence that social skills of older children (i.e., adolescents) with RASopathies were more discrepant from their peers than social skills of younger children with RASopathies.

A higher proportion of the children with NF1 in this sample had a parent who was confirmed to be affected with the same condition (44%) as compared with children with NS (10%). In the sibling group, 16% had a parent affected with either NS or NF1 (Table 1). In each group, there were additional cases in which a diagnosis was suspected but not confirmed in the parent. To examine whether being rated by a parent who also had a RASopathy was likely to impact our results (i.e., resulting in higher or lower symptom ratings for those children), we examined ratings for this subset of participants. Ratings of social skills were provided by a parent affected by a RASopathy for three children with NS, seven children with NF1, and three unaffected siblings. Within the NF1 group, there was no significant difference in the social skills outcomes for children who were rated by a parent who also had NF1 as compared to children rated by a parent who was not affected [mean difference = − 3.7; 95% CI: (− 8.7, 16.0), *p* = 0.796]. There were also no significant differences in ratings on any of the predictor variables. This comparison was not examined within the NS or sibling groups, given the small number of children being rated by an affected parent in these groups.

### Predictors of social skills

**Fig. 2:**
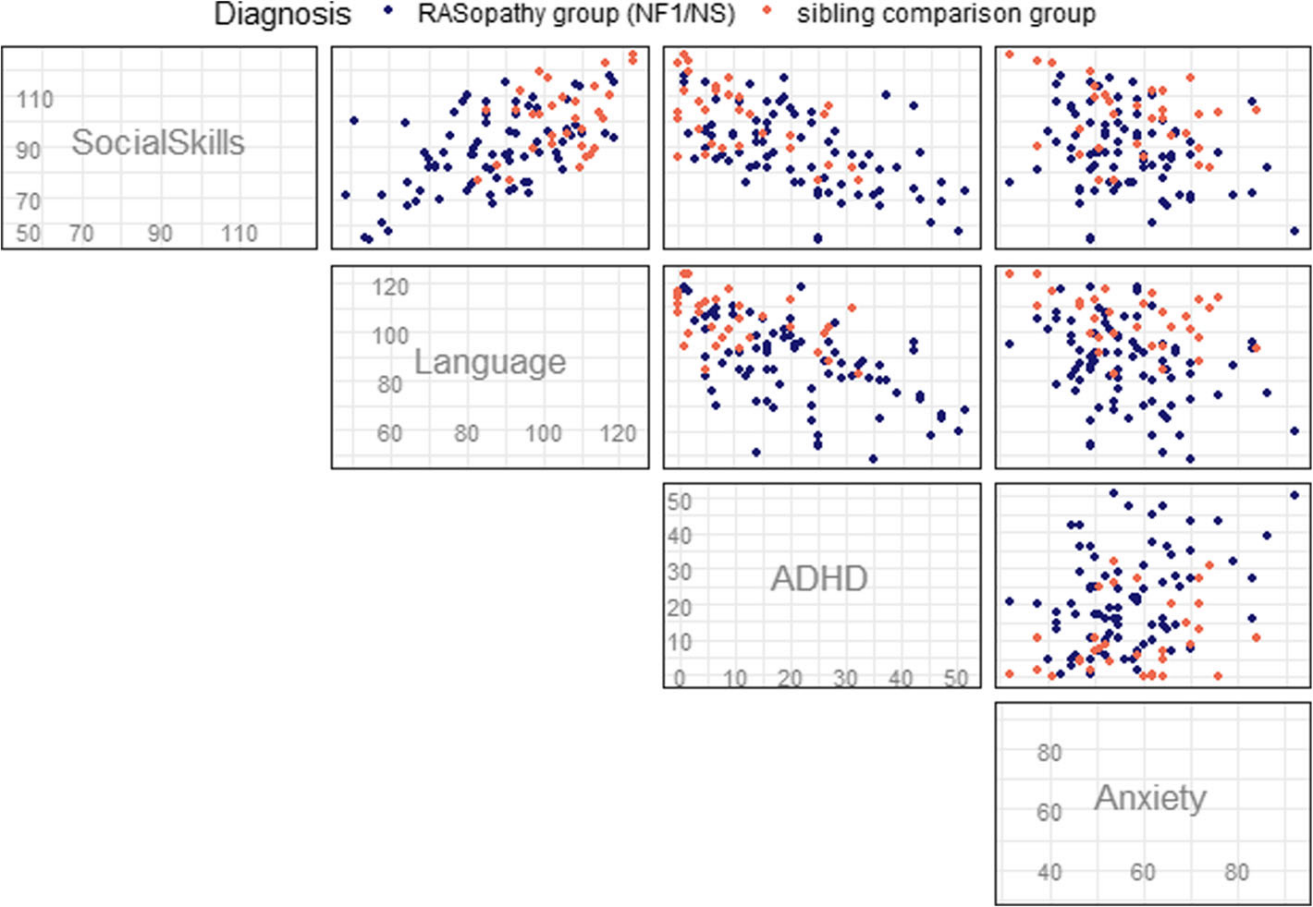
Pairwise scatterplots of the relationships between the neuropsychological variables and social skills in children with RASopathies and unaffected siblings. Legend: Tests measuring each domain: Social Skills = Social Skills Improvement System (SSIS), Social Skills Composite; Language = Children’s Communication Checklist, Second Edition (CCC-2), General Communication Composite; ADHD = ADHD Rating Scale, Fourth Edition (ADHD-RS), Home Version; Anxiety = Behavior Assessment Scale for Children, Second Edition (BASC-2), Anxiety subscale

Pairwise scatterplots (Fig. 2) show the associations between children’s scores on the primary outcome variable (SSIS Social Skills) and their scores on the measures of neuropsychological functioning (language ability, ADHD symptoms and anxiety). Children without a RASopathy diagnosis generally exhibited fewer neuropsychological challenges across these measures (e.g., better language skills, fewer ADHD symptoms).

Linear regression models to identify neuropsychological predictors of social skills are presented in Table 4. To identify the most sensitive language component, pragmatic language and structural language were considered separately. Results showed that pragmatic language abilities and ADHD symptoms were strong predictors of social skills, but structural language and anxiety symptoms were not. In the full cohort model, one additional point on the pragmatic language subscale was associated with an expected increase of 0.77 SSIS points, while one additional point on the ADHD scale was associated with an expected decrease of 0.37 SSIS points.

**Table 4:** Coefficients from linear regression models to predict social skills (SSIS)

	NS + NF1 only (*n* = 78)		NS + NF1 + Siblings (*n* = 110)	
Variable	Coefficient	95% CI	Coefficient	95% CI
Intercept	76.20	(56.12, 96.28)	78.35	(61.79, 94.90)
CCC-2 Pragmatic Subscale	0.71	(0.29, 1.13)	0.77	(0.41, 1.13)
CCC-2 Structural Subscale	− 0.09	(− 0.46, 0.28)	− 0.14	(− 0.46, 0.17)
ADHD-RS (Total)	− 0.32	(− 0.57, − 0.07)	− 0.37	(− 0.58, − 0.16)
BASC-2 Anxiety scale	0.01	(− 0.25, 0.26)	− 0.01	(− 0.21, 0.18)

Coefficients estimate change in expected outcome per unit of each continuous variable

Notably, the relationship of the predictors to the social skills outcome did not meaningfully change with the addition of the unaffected sibling group. Thus, while children with NS or NF1 tended to have lower scores on pragmatic language and social skills measures and higher scores on ADHD symptom ratings relative to unaffected siblings, the functional relationship of these variables was similar in each of the different populations, consistent with our pairwise scatterplots (Fig. 2). We also evaluated whether the relationship between neuropsychological predictors and social skills varied by RASopathy diagnosis (NF1 vs. NS vs. unaffected siblings) by adding interaction terms (diagnosis with each predictor) to the regression model described above. None of the interaction terms were statistically significant (*F* test for all NF1 vs NS interaction terms *p* = 0.43; *F* test for all unaffected sibling interaction terms *p* = 0.71). This was again consistent with our analysis in Table 4 and Fig. 2, indicating that the neuropsychological variables had similar associations with social skills across the diagnostic groups.

## Discussion

Social skills facilitate the ability of children to develop meaningful interpersonal relationships. As such, they can have a major impact on the psychological well-being of children and adolescents with neurodevelopmental disorders, as well as on longer-term academic and employment outcomes [37, 38]. Recent research has drawn attention to the heightened risk for social impairment among children with RASopathies, with some estimates as high as 1 in 3 children with NS and NF1 experiencing a degree of social impairment similar to children who have a diagnosis of ASD (Table 2). Given these striking findings, there is an important need to improve our understanding of the factors that underlie social dysfunction among children affected by these conditions. Increasing the ability of clinicians and educators to predict social skills deficits and to support effective social functioning is critical to optimizing patient care and improving outcomes for children with RASopathies.

Our study confirms previous findings [12, 13] indicating overall weaker social skills in children with RASopathies relative to same-aged peers who do not have RASopathies. The fact that the comparison group in this study was comprised of unaffected individuals from the same families (i.e., siblings) further strengthens the notion that biological alterations in these syndromes can play a key role in development of social behavior. Our results also suggest that parent insight regarding their child’s social skills was similar whether or not the parent also had a RASopathy. Although the number of participants included in this study was too small to assess the matter comprehensively, parent ratings were generally consistent in the NF1 group whether or not the parent providing these ratings was also affected with NF1.

Expanding on previous research, the cross-syndrome comparison presented here demonstrates that the prevalence and severity of challenges related to social competence appears to be remarkably similar in children with NS as compared to those with NF1 (Fig. 1). Other investigators have also noted striking similarities in these two conditions with regard to certain neurocognitive phenotypes, such as marked ADHD symptomatology [9]. Such observations raise the intriguing question of whether shared pathophysiological mechanisms may underlie the neuropsychological concerns that are described in NS and NF1. Although research using mouse models has suggested that distinct mechanisms of neurotransmitter disruption (e.g., increased excitatory vs. inhibitory synaptic transmission) may occur in each of these two conditions [39], the observed phenotypic similarities suggest that dysregulated molecular signaling in both syndromes may ultimately impact the neurodevelopmental process in similar ways.

Despite substantial differences in overall social skills between the clinical groups and the sibling comparison group, it is notable that for more than half of children in the NS and NF1 groups (56–59%), social skills ratings were within the average range, indicating that most parents did not perceive their child’s social skills to be highly problematic. This finding is of particular interest in light of previous research showing that parent ratings of social skills and social communication of children with NF1 tend to indicate greater difficulties relative to teacher and self-ratings [13, 14, 40]. The fact that many of the children with RASopathies were rated as having adequate social skills in this study, even by caregivers who are most likely to be sensitive to their child’s difficulties, underscores the wide variability in the phenotype of both NS and NF1 as well as the potential for substantial neurobehavioral resilience among some affected individuals.

Similarly, a relatively small percentage of children in the RASopathy groups (15% for NS and 5% for NF1) scored in the severely impaired range (SS ≤ 70) on the SSIS. This finding suggests that observed group differences in social skills compared with the unaffected sibling group may largely be driven by the presence of moderate difficulties in a subset of children in the clinical groups, rather than by large numbers of patients with severely impaired social skills. Similar findings have been reported in studies of RASopathies involving assessment of ASD symptomatology. In these studies, there is often a substantial group of children who do not meet full criteria for ASD but who present with sub-clinical symptoms (e.g., weaker social awareness, inflexibility, poor self-regulation) [20, 41]. Thus, our findings add to a growing body of research highlighting the presence of a relatively large subset of children with NS and NF1 who have mild-to-moderate social difficulties but are unlikely to be classified as having ASD. While these children may not require the same intensity of intervention as patients with more severe, pervasive social impairment, social weaknesses may impact academic performance, well-being and quality of relationships. As such, there is a great need to validate methods of intervention (e.g., social skills groups, behavioral therapies, parent coaching, telehealth interventions, support group workshops) that may be used to support improved social interactions among children with RASopathies who do not require or qualify for comprehensive ASD services.

Importantly, recruitment and assessment methods are likely to play a significant role in estimates of prevalence of social impairment among RASopathy cohorts. For example, a recent study using multidisciplinary diagnostic assessment methods to assess an unselected cohort of individuals with NF1 yielded a significantly lower estimate of ASD prevalence (10.9%) [21] relative to studies that have evaluated this population using only screening methods or that have selected probands based upon an ASD presumption (i.e., a high score on screening instruments). This finding suggests that a higher prevalence and degree of social impairment is likely to be observed among patients referred to specialty clinics due to established concern for a presenting problem, as compared to clinics that routinely assess all patients with NS or NF1 as standard of care.

In terms of identifying neuropsychological variables that may be associated with social competence in this population, the current study did not directly examine the relationship between global measures of cognitive ability (e.g., IQ, achievement) and social skill development, given the relatively weak relationship of these more global measures to social competence in previous studies of NF1 (e.g., [12, 13]). These reports are consistent with our finding that only one of the eight study participants who severely impaired social skills had been given a diagnosis of intellectual disability, and that only one of the six patients with identified intellectual disability had severely impaired social skills. While this evidence does not imply that cognitive ability is completely uncoupled with social competence, it suggests that examination of more specific domains of cognitive or neuropsychological function may be a more fruitful method of identifying potential underlying neuropsychological correlates of social impairment than examining intellectual ability more broadly [42].

Communication and language skills are an area of significant weakness for some children with RASopathies. Many children with NF1 or NS have speech articulation difficulties, and approximately 30% of children with NS [22] and 23–37% of children with NF1 [24, 43] demonstrate impairment on gold-standard measures of language functioning. Parent rating measures indicate that children with NS are at increased risk for pragmatic communication problems relative to unaffected children [22, 23] including skills such as interpreting gestures and facial expressions, understanding conversational conventions and making inferences about meaning in language based on context. On more direct, objective measures, individuals with NS or NF1 have been found to have more difficulties than unaffected peers with regard to perception and identification of emotional expression in faces [44–47].

Our study highlights the importance of communication deficits, and primarily social language deficits, as a key predictor of social competence among children with RASopathies in daily life settings. While overall communication skills were significantly correlated with social skills, our regression analysis indicated that difficulties with social-pragmatic language were a strong predictor of social skills deficits in both patient groups as well as in the unaffected sibling group. Structural aspects of language such as speech and syntax demonstrated a less robust association to social competence. Thus, measures used to diagnose developmental language delay may be a less sensitive predictor of impaired social functioning in patients with RASopathies compared to measures specifically designed to assess pragmatic aspects of language. Future research should examine whether caregiver report measures such as the CCC-2 or direct testing of pragmatic language can better identify social language dysfunction in individuals with RASopathies. While direct testing allows for a more objective characterization of language development, caregiver report measures have the advantage that they are completed by someone who knows the child well, they may be less sensitive to day-to-day fluctuations in performance, and they can assess a wide range of pragmatic abilities in one measure, including developmentally atypical behaviors that might be difficult to elicit in a test situation [30].

Given that idiopathic ADHD is associated with deficits in social competence [48], some research has explored the relationship between social dysfunction and ADHD symptoms in individuals with RASopathies. This research has produced conflicting findings. Several studies have reported an association between parent ratings assessing ADHD symptoms (e.g., inattention, hyperactivity, impulsivity) and measures of social competence [13, 46] or ASD symptomatology [20, 41] in children with NF1. In contrast, Lewis and colleagues failed to find a relationship between ADHD symptomatology and social competence in a cohort of children with NF1 [12]. Similarly, in a study involving children with NS, individuals with severe or mild ASD symptoms did not differ from children without ASD symptoms in terms of parent and teacher ratings of inattention and hyperactivity [20]. Thus, the true impact of ADHD symptoms on social functioning has been difficult to assess. In our cohort, all eight of the participants with severely impaired social skills had been previously diagnosed as having ADHD. Furthermore, ADHD symptoms were a significant predictor of social skills in both the NS and NF1 groups, even when holding pragmatic language skills constant. This finding demonstrates that ADHD symptomatology is associated with social challenges not only in NF1, but in other RASopathies as well.

Another comorbid condition that can contribute to social difficulties is anxiety. Social anxiety is frequently found to be a contributor to social challenges in children with genetic disorders (e.g., fragile X syndrome [16]), and a recent study found a relationship between social competence and social anxiety in children with NF1 [12]. In the present study, we did not observe an association between social skills and symptoms of anxiety (e.g., frequency of worrying, nervousness, panic symptoms). While this finding does not rule out the possibility that some children with RASopathies have social withdrawal due to anxiety, it suggests that generalized anxiety is not likely a good explanation for the observed social skills deficits in the patient groups relative to their unaffected siblings. An interesting consideration is that it may be challenging for caregivers and other observers to identify whether withdrawn behaviors reflect underlying anxiety or whether these behaviors are more indicative of ASD (i.e., an underlying deficit in social reciprocity). Distinguishing socially anxious behavior from more pervasive developmental impairment may require more comprehensive diagnostic assessments, including behavioral and/or psychophysiological measures.

### Limitations

Several methodological limitations should be considered relative to the current study. First, the study relied on parent report measures and did not provide perspective from the children and adolescents themselves. In terms of our parent ratings of social skills, there were fewer patients in the NS group that had an affected parent relative to the NF1 group, making it difficult to ascertain whether parents who also have the same genetic syndrome may have a different perspective than parents who are unaffected. Second, there was no objective measurement of the construct of social competence (i.e., observation of social skills or peer interaction), or of cognitive abilities (i.e., IQ or direct language testing). Future research using clinic-based (rather than parent observation) of these constructs may lead to additional insights regarding social-emotional function in RASopathies. Finally, not every patient had genetic confirmation of the condition; due to lack of insurance coverage for genetic testing among some health care plans and the fact that gene testing does not yield a positive result for all children with diagnostic features of NS, some children were included in this study despite having only a clinical diagnosis by a geneticist experienced in the diagnosis of NS. Therefore, there is a small possibility of inclusion of children in this sample who were incorrectly diagnosed. It was also not feasible to perform analysis of genotype-phenotype correlations given the small sample size.

## Conclusions

The discovery that the genetic mechanisms causing NS and NF1 result in dysregulation of a common molecular pathway explains the fact that there are overlapping aspects of the phenotype when it comes to chronic disease symptoms such as cardiac disease, growth and endocrine issues, skin features and increased risk for malignancies. Both syndromes are also associated with significant risk for neurocognitive and behavioral comorbidities. Difficulties with social skill development have been previously described in children with NF1 and other RASopathies and were confirmed by the present study. Despite the group differences we observed, it is also notable that the majority of participants with NS and NF1 in this study did not have severe social skills impairment. This finding underscores the fact that social impairment is not consistently associated with NS or NF1, reflecting the considerable variability in neurodevelopmental sequelae that occur within these conditions. Nevertheless, for the sizeable minority of children with these conditions who are impacted by social challenges, prompt evaluation and intervention may substantially improve long-term academic and career trajectory as well as quality of life.

In terms of understanding the etiology of neurodevelopmental challenges in RASopathies, there is increasing evidence that such challenges may arise due to altered neurodevelopmental processes that are underway even prior to birth, and that these biological mechanisms may be more important than medical variables (e.g., cardiac disease, treatment effects) in explaining the variation in neurodevelopmental function [8, 9, 49]. Neuroimaging studies showing reduced white matter integrity and atypical patterns of functional connectivity within brain networks of individuals with NF1 suggest plausible neurobiological explanations for social behavioral differences [50, 51]. Similar markers of neurobiological structure and function should also be investigated in studies of NS. Indeed, our findings suggest that similar underlying neuropsychological mechanisms are associated with the social skills deficits seen in children with both RASopathies. In particular, the severity of ADHD symptoms and pragmatic language difficulties in the children with RASopathies in this study was related to the degree of social skill deficit. These two areas (social language development and ADHD symptoms) may therefore represent important targets for interventions for children with RASopathies who are at risk for social difficulties.

## References

[1] Rauen KA (2013). The RASopathies. Annu Rev Genomics Hum Genet.

[2] Mendez HM, Opitz JM (1985). Noonan syndrome: a review. Am J Med Genet.

[3] Harms FL, Alawi M, Amor DJ, Tan TY, Cuturilo G, Lissewski C, Brinkmann J, Schanze D, Kutsche K, Zenker M (2018). The novel RAF1 mutation p.(Gly361Ala) located outside the kinase domain of the CR3 region in two patients with Noonan syndrome, including one with a rare brain tumor. Am J Med Genet A.

[4] Aoki Y, Niihori T, Inoue S, Matsubara Y (2016). Recent advances in RASopathies. J Hum Genet.

[5] Tartaglia M, Gelb BD, Zenker M (2011). Noonan syndrome and clinically related disorders. Best Pract Res Clin Endocrinol Metab.

[6] Williams VC, Lucas J, Babcock MA, Gutmann DH, Korf B, Maria BL (2009). Neurofibromatosis type 1 revisited. Pediatrics.

[7] Adviento B, Corbin IL, Widjaja F, Desachy G, Enrique N, Rosser T, Risi S, Marco EJ, Hendren RL, Bearden CE (2014). Autism traits in the RASopathies. J Med Genet.

[8] Vogel AC, Gutmann DH, Morris SM (2017). Neurodevelopmental disorders in children with neurofibromatosis type 1. Dev Med Child Neurol.

[9] Green T, Naylor PE, Davies W (2017). Attention deficit hyperactivity disorder (ADHD) in phenotypically similar neurogenetic conditions: Turner syndrome and the RASopathies. J Neurodev Disord.

[10] Alfieri P, Piccini G, Caciolo C, Perrino F, Gambardella ML, Mallardi M, Cesarini L, Leoni C, Leone D, Fossati C (2014). Behavioral profile in RASopathies. Amer J Med Genet Part A.

[11] Pierpont EI, Tworog-Dube E, Roberts AE (2015). Attention skills and executive functioning in children with Noonan syndrome and their unaffected siblings. Dev Med Child Neurol.

[12] Lewis AK, Porter MA, Wiliams TA, North KN, Payne JM (2016). Social competence in children with neurofibromatosis type 1: relationships with psychopathology and cognitive ability. J Child Dev Disord.

[13] Barton B, North K (2004). Social skills of children with neurofibromatosis type 1. Dev Med Child Neurol.

[14] Noll RB, Reiter-Purtill J, Moore BD, Schorry EK, Lovell AM, Vannatta K, Gerhardt CA (2007). Social, emotional, and behavioral functioning of children with NF1. Am J Med Genet Part A.

[15] de Bildt A, Serra M, Luteijn E, Kraijer D, Sytema S, Minderaa R (2005). Social skills in children with intellectual disabilities with and without autism. J Intellect Disabil Res.

[16] Reisinger DL, Roberts JE (2017). Differential relationships of anxiety and autism symptoms on social skills in young boys with fragile X syndrome. Am J Intellect Dev Disabil.

[17] Hyman SL, Shores A, North KN (2005). The nature and frequency of cognitive deficits in children with neurofibromatosis type 1. Neurology.

[18] Pierpont EI (2016). Neuropsychological functioning in individuals with Noonan syndrome: a systematic literature review with educational and treatment recommendations. J Ped Neuropsychol.

[19] Garg S, Green J, Leadbitter K, Emsley R, Lehtonen A, Evans DG, Huson SM (2013). Neurofibromatosis type 1 and autism spectrum disorder. Pediatrics.

[20] Garg S, Brooks A, Burns A, Burkitt-Wright E, Kerr B, Huson S, Emsley R, Green J (2017). Autism spectrum disorder and other neurobehavioural comorbidities in rare disorders of the Ras/MAPK pathway. Dev Med Child Neurol.

[21] Eijk S, Mous SE, Dieleman GC, Dierckx B, Rietman AB, de Nijs PFA, Ten Hoopen LW, van Minkelen R, Elgersma Y, Catsman-Berrevoets CE (2018). Autism spectrum disorder in an unselected cohort of children with Neurofibromatosis Type 1 (NF1). J Autism Dev Disord.

[22] Pierpont EI, Ellis Weismer S, Roberts AE, Tworog-Dube E, Pierpont ME, Mendelsohn NJ, Seidenberg MS (2010). The language phenotype of children and adolescents with Noonan syndrome. J Speech Lang Hear Res.

[23] Selas M, Helland WA (2016). Pragmatic language impairment in children with Noonan syndrome. Clin Linguist Phon.

[24] Brei NG, Klein-Tasman BP, Schwarz GN, Casnar CL (2014). Language in young children with neurofibromatosis-1: relations to functional communication, attention, and social functioning. Res Dev Disabil.

[25] National Institutes of Health Consensus Development Conference Statement: Neurofibromatosis. Bethesda, Md., USA, July 13-15, 1987. Neurofibromatosis 1988;1:172–178. 3152465

[26] Gresham F, Elliott S (2008). Social skills improvement system.

[27] Bishop DV (2006). Children's Communication Checklist, Second Edition, U.S. Edition.

[28] Philofsky A, Fidler DJ, Hepburn S (2007). Pragmatic language profiles of school-age children with autism spectrum disorders and Williams syndrome. Am J Speech Lang Pathol..

[29] Timler GR (2014). Use of the Children’s Communication Checklist-2 for classification of language impairment risk in young school-age children with attention-deficit/hyperactivity disorder. Am J Speech Lang Pathol.

[30] Volden J, Phillips L (2010). Measuring pragmatic language in speakers with autism spectrum disorders: comparing the Children’s Communication Checklist--2 and the Test of Pragmatic Language. Am J Speech Lang Pathol..

[31] Lew AR, Lewis C, Lunn J, Tomlin P, Basu H, Roach J, Rakshi K, Martland T (2015). Social cognition in children with epilepsy in mainstream education. Dev Med Child Neurol.

[32] DuPaul G, Power T, Anastopoulos A, Reid R (1998). ADHD rating scale IV.

[33] American Psychiatric Association: Diagnostic and statistical manual of mental disorders, Fifth Edition. Arlington: American Psychiatric Publishing; 2013.

[34] Reynolds C, Kamphaus R: Behavior Assessment System for Children, Second Edition. Circle Pines: American Guidence Service; 2004.

[35] Young O, Perati S, Weiss LA, Rauen KA (2018). Age and ASD symptoms in Costello syndrome. Am J Med Genet A.

[36] Kolesnik AM, Jones EJH, Garg S, Green J, Charman T, Johnson MH, Team E-B (2017). Early development of infants with neurofibromatosis type 1: a case series. Mol Autism.

[37] Hotton M, Coles S (2016). The effectiveness of social skills training groups for individuals with autism Spectrum disorder. Rev J Autism Dev Disord.

[38] Carter EW, Austin D, Trainor AA (2012). Predictors of postschool employment outcomes for young adults with severe disabilities. J Disabil Policy Stu.

[39] Costa-Mattioli M (2014). ERKquake in Noonan syndrome: one step closer to personalized medicine. Nat Neurosci.

[40] Garg S, Lehtonen A, Huson SM, Emsley R, Trump D, Evans DG, Green J (2013). Autism and other psychiatric comorbidity in neurofibromatosis type 1: evidence from a population-based study. Dev Med Child Neurol.

[41] Walsh KS, Velez JI, Kardel PG, Imas DM, Muenke M, Packer RJ, Castellanos FX, Acosta MT (2013). Symptomatology of autism spectrum disorder in a population with neurofibromatosis type 1. Dev Med Child Neurol.

[42] Huijbregts SC, de Sonneville LM (2011). Does cognitive impairment explain behavioral and social problems of children with neurofibromatosis type 1?. Behav Genet.

[43] Thompson HL, Viskochil DH, Stevenson DA, Chapman KL (2010). Speech-language characteristics of children with neurofibromatosis type 1. Am J Med Genet A.

[44] Wingbermühle E, Egger JI, Verhoeven WM, van der Burgt I, Kessels RP (2012). Affective functioning and social cognition in Noonan syndrome. Psychol Med.

[45] Lewis AK, Porter MA, Williams TA, Bzishvili S, North KN, Payne JM (2017). Facial emotion recognition, face scan paths, and face perception in children with neurofibromatosis type 1. Neuropsychology.

[46] Allen T, Willard VW, Anderson LM, Hardy KK, Bonner MJ (2016). Social functioning and facial expression recognition in children with neurofibromatosis type 1. J Intellect Disabil Res.

[47] Huijbregts S, Jahja R, De Sonneville L, de Breij S, Swaab-Barneveld H (2010). Social information processing in children and adolescents with neurofibromatosis type 1. Dev Med Child Neurol.

[48] Julvez J, Forns M, Ribas-Fito N, Torrent M, Sunyer J (2011). Attention behavior and hyperactivity and concurrent neurocognitive and social competence functioning in 4-year-olds from two population-based birth cohorts. Eur Psychiatry.

[49] Pierpont EI, Pierpont ME, Mendelsohn NJ, Roberts AE, Tworog-Dube E, Seidenberg MS (2009). Genotype differences in cognitive functioning in Noonan syndrome. Genes Brain Behav.

[50] Loitfelder M, Huijbregts SC, Veer IM, Swaab HS, Van Buchem MA, Schmidt R, Rombouts SA (2015). Functional connectivity changes and executive and social problems in neurofibromatosis type I. Brain Connect.

[51] Koini M, Rombouts S, Veer IM, Van Buchem MA, Huijbregts SCJ (2017). White matter microstructure of patients with neurofibromatosis type 1 and its relation to inhibitory control. Brain Imaging Behav.

[52] Richards C, Jones C, Groves L, Moss J, Oliver C (2015). Prevalence of autism spectrum disorder phenomenology in genetic disorders: a systematic review and meta-analysis. Lancet Psychiatry.

[53] Plasschaert E, Descheemaeker MJ, Van Eylen L, Noens I, Steyaert J, Legius E (2015). Prevalence of autism spectrum disorder symptoms in children with neurofibromatosis type 1. Am J Med Genet B Neuropsychiatr Genet.

[54] Perrino F, Licchelli S, Serra G, Piccini G, Caciolo C, Pasqualetti P, Cirillo F, Leoni C, Digilio MC, Zampino G (2018). Psychopathological features in Noonan syndrome. Eur J Paediatr Neurol.

[55] Pride NA, Payne JM, North KN (2012). The impact of ADHD on the cognitive and academic functioning of children with NF1. Dev Neuropsychol.

